# Clinical Validation of Multiparametric Ultrasound for Detecting Clinically Significant Prostate Cancer Using Computer-Aided Diagnosis: A Direct Comparison with the Magnetic Resonance Imaging Pathway

**DOI:** 10.1016/j.euros.2024.06.012

**Published:** 2024-07-01

**Authors:** Daniel L. van den Kroonenberg, Auke Jager, Anna Garrido-Utrilla, Johannes B. Reitsma, Arnoud W. Postema, Harrie P. Beerlage, Jorg R. Oddens

**Affiliations:** a Department of Urology, Amsterdam UMC, Amsterdam, The Netherlands; b Clinical Data Collection, Angiogenesis Analytics, Den Bosch, The Netherlands; c Julius Center for Health Sciences and Primary Care, UMC Utrecht, Utrecht, The Netherlands; d Department of Urology, Leids Universitair Medisch Centrum, Leiden, The Netherlands

**Keywords:** Prostate cancer, Magnetic resonance imaging, Ultrasound, Artificial intelligence, Computer-aided diagnosis, Prostate biopsy, Targeted biopsy

## Abstract

We present the protocol for a study testing the hypothesis that a computer-aided diagnosis (CAD) system for three-dimensional multiparametric ultrasound (3D mpUS) is noninferior to magnetic resonance imaging (MRI) in guiding prostate biopsies for detection of clinically significant prostate cancer (csPCa). The prospective study has a fully paired design for assessment of diagnostic accuracy and is registered on ClinicalTrials.gov as NCT06281769. A total of 438 biopsy-naïve men scheduled for prostate MRI evaluation because of an abnormal digital rectal examination and/or elevated serum prostate-specific antigen will be included. All patients will undergo both MRI (multiparametric or biparametric) and 3D mpUS with CAD (PCaVision). Suspicious lesions will be independently identified using each imaging technique. MRI targeted biopsy (TBx) and/or PCaVision TBx will be performed if suspicious lesions are identified on imaging. When both PCaVision and MRI identify lesions in an individual patient, the TBx order for this patient will be randomized. Three TBx samples per lesion will be taken for a maximum of two lesions per modality. The primary objective is the detection rate for csPCa (International Society of Urological Pathology grade group [GG] ≥2) with the PCaVision versus the MRI TBx pathway. The noninferiority margin for the absolute difference in detection rates is set at a difference of 5%. Secondary outcomes are the proportion of men in whom TBx could have been safely omitted in each pathway. Additional diagnostic accuracy analyses will be performed for different definitions of PCa (GG ≥3; GG ≥2 with cribriform growth and/or intraductal carcinoma; and GG 1). The frequency of insufficient image quality for the two pathways will also be assessed. Lastly, we will determine the diagnostic performance for csPCa detection at various 3D mpUS image quality thresholds for PCaVision.

## Take Home Message

This clinical validation trial will provide evidence on the clinical performance of a computer-aided diagnosis pathway for prostate cancer, the PCaVision™ pathway, in comparison to the standard magnetic resonance imaging pathway.

## 1 Introduction and hypotheses

The incidence of prostate cancer (PCa) has risen, making it the second most common malignancy among men worldwide, with approximately 1.4 million new cases in 2020 [1]. As the population ages, the economic burden of PCa is expected to increase [2], [3]. Accurate assessment of clinically significant tumors and their risk of progression is crucial for optimal PCa management to prevent overtreatment of low-risk cases and ensure timely intervention for those with clinically significant PCa (csPCa) [4], [5].

The European Association of Urology (EAU) guidelines currently recommend magnetic resonance imaging (MRI) as the preferred imaging method for PCa [6]. This recommendation is based on three multicenter trials that demonstrated that MRI targeted biopsy (TBx) reduces the number of unnecessary biopsies and identifies more csPCa in comparison to transrectal ultrasound-guided systematic biopsy (SBx) [7], [8], [9]. Nevertheless, the pooled sensitivity and specificity of MRI for csPCa detection vary across studies because of differences in equipment, study design, and interobserver variability [10], [11], [12], [13], [14], [15]. Furthermore, the increasing demand for MRI scanners and skilled radiologists is placing pressure on capacity, resulting in logistic inefficiencies in radiology and biopsy processes rather than a streamlined approach.

Ultrasound (US) could be a more accessible and cost-effective imaging option in comparison to MRI for PCa diagnosis. While US is primarily used in guiding prostate biopsies, advances such as contrast-enhanced US (CEUS), micro-US, and shear wave elastography (SWE) have enhanced diagnostic capabilities [9], [16], [17], [18].

CEUS has shown promising sensitivity (91%) but limited specificity (56%) in this setting [16]. To improve its diagnostic value, quantitative interpretation algorithms such as contrast-US-dispersion imaging (CUDI) have been developed [16], [19], [20], [21]. However, two-dimensional (2D) CEUS lacks clinical viability because of its dependence on operator expertise and limited imaging plane. By contrast, three-dimensional (3D) CEUS offers a significant improvement, allowing quick acquisition with a single contrast bolus and providing accurate native 3D imaging without the need for complex reconstructions of 2D planes. Despite its potential, interpretation of 3D CEUS images remains challenging and operator-dependent.

Computer-aided diagnosis (CAD) is capable of overcoming these interpretation challenges. A CAD system (PCaVision; Angiogenesis Analytics, Den Bosch, Netherlands) based on multiparametric US (mpUS, including 3D CEUS, 3D SWE, and 3D B-mode) that uses artificial intelligence (AI) has been developed. The system has been trained on a comprehensive data set for more than 600 patients, including both confirmed PCa cases (undergoing prostatectomy) and negative cases (Prostate Imaging-Reporting and Data System [PI-RADS] score ≤2 or negative SBx). The PCaVision CAD system includes an automated quality assessment of 3D mpUS images. If an image meets the quality threshold, the system generates a heat map indicating the probability of csPCa presence.

The current standard diagnostic pathway for PCa involves MRI followed by TBx if suspicious lesions are detected. An alternative would be a PCaVision CAD pathway, in which 3D mpUS imaging with PCaVision analysis is followed by TBx in cases with suspicious lesions. The overall aim of this study is to compare the diagnostic accuracy of these two diagnostic pathways for detection of csPCa in biopsy-naïve men.

### 1.1 Primary objective

The primary objective is to demonstrate noninferiority of the PCaVision pathway to the standard MRI-based pathway for detection of csPCa (defined as International Society of Urological Pathology [ISUP] grade group [GG] ≥2).

### 1.2 Secondary objectives

There are a number of secondary study objectives. The first is to compare the proportion of men for whom TBx could be safely omitted in the PCaVision pathway versus the MRI pathway. This is defined as the number of men in whom no lesions for TBx were identified via PCaVision and no csPCa was detected via either MRI TBx or SBx. The combined findings for all biopsy types serve as the reference in defining “safe”.

The second is to perform the same diagnostic accuracy analyses as described for the primary objectives but using different definitions of PCa. These include: (1) GG ≥3 in any of the biopsy cores taken from a lesion; (2) GG ≥2 with cribriform growth and/or intraductal carcinoma in any of the biopsy cores taken from a lesion; and (3) GG 1.

The third secondary objective is to compare the number of cases with insufficient image quality between the 3D mpUS and MRI modalities. Insufficient 3D mpUS image quality is defined by the PCaVision software for a fixed quality threshold. Insufficient MRI quality is defined by the urologist as images that are unusable for PCa diagnosis or for performing TBx.

The final secondary objective is to perform the same diagnostic accuracy analyses using various image quality thresholds for 3D mpUS, as set by the PCaVision software.

## 2 Study design

The study population consists of biopsy-naïve men with suspected PCa on the basis of an abnormal digital rectal examination or elevated serum prostate-specific antigen (PSA). Patients will be included in the study if they are scheduled for prostate MRI evaluation.

Exclusion criteria are mostly related to MRI, the contrast agent used for CEUS imaging, and the biopsy procedure. Table 1 lists all the inclusion and exclusion criteria.

**Table 1:** Eligibility criteria

**Inclusion criteria**	
1.	Age ≥18 yr
2.	Biopsy-naïve
3.	Clinical suspicion of prostate cancer
4.	Scheduled for prostate MRI evaluation because of a suspicious digital rectal examination and/or elevated serum prostate-specific antigen
5.	Signed informed consent
**Exclusion criteria**	
1.	Active (urinary tract) infection or prostatitis
2.	History of a cardiac right-to-left shunt
3.	Allergic to sulfur hexafluoride or any of the other ingredients of the ultrasound contrast agent (SonoVue)
4.	Current treatment with dobutamine
5.	Severe pulmonary hypertension (pulmonary artery pressure >90 mm Hg), uncontrolled systemic hypertension, or respiratory distress syndrome
6.	Any (further) contraindication to MRI or three-dimensional multiparametric ultrasound imaging
7.	History of prostate surgery
8.	Current 5α-reductase inhibitor use for at least 3 mo
9.	Incapable of understanding the language in which the patient information is given

MRI = magnetic resonance imaging.

## 3 Protocol overview

**Fig. 1:**
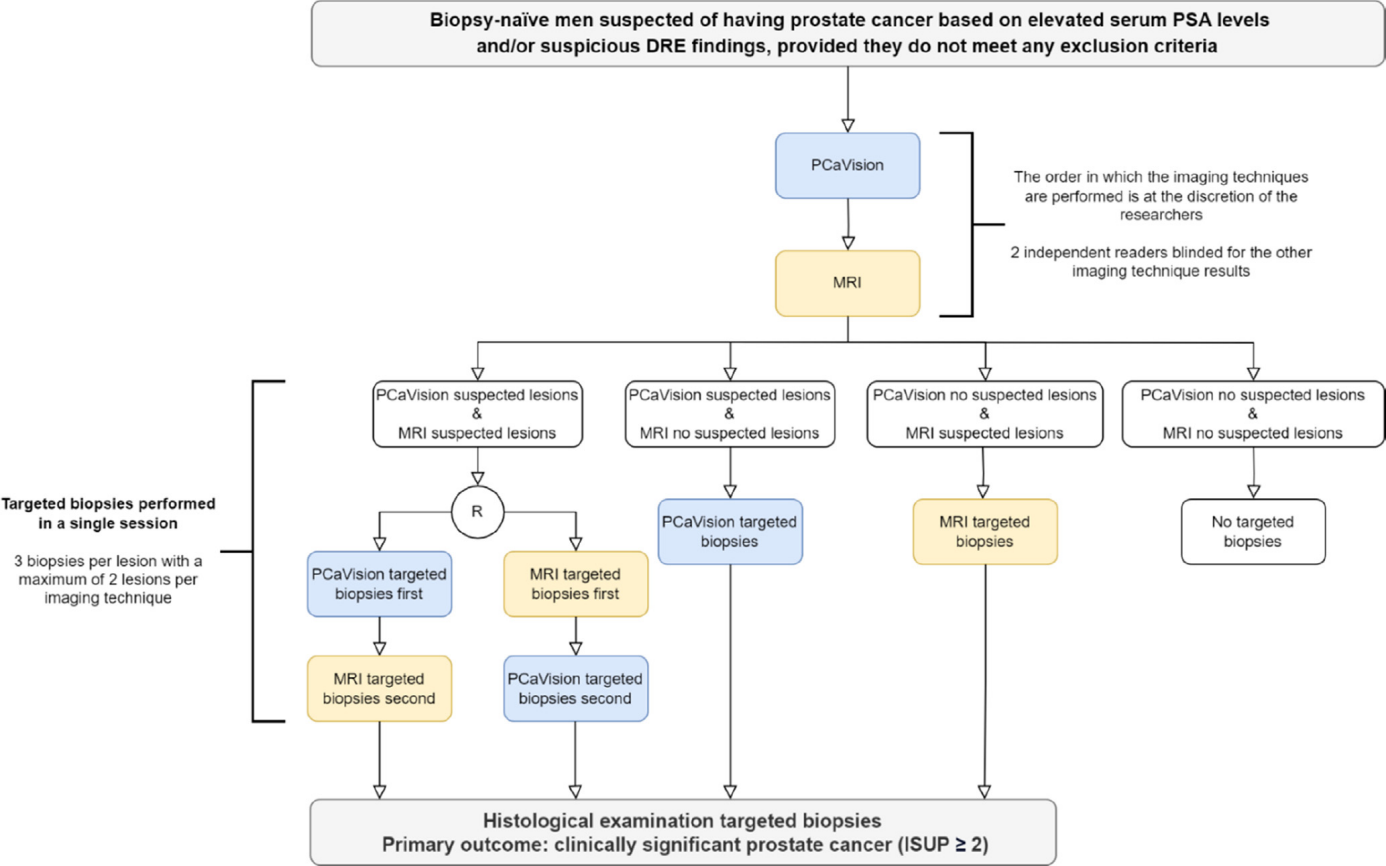
Study flowchart. PSA = prostate-specific antigen; DRE = digital rectal examination; ISUP: International Society of Urological Pathology; R = randomization.

This is a prospective study evaluating diagnostic accuracy via a fully paired design for biopsy-naïve men suspected of having PCa. A flowchart illustrating the design is presented in Figure 1. In summary, all patients will undergo two imaging procedures (MRI and 3D mpUS with PCaVision). Suspicious lesions will be independently identified via each imaging modality by readers blinded to the results from the other imaging modality. If suspicious lesions are identified, three targeted biopsy cores will be taken per lesion for a maximum of two lesions per imaging modality. If three or more suspicious lesions are identified on MRI, two lesions will be selected on the basis of (1) PI-RADS score and (2) lesion size. If three or more suspicious lesions are identified on PCaVision, two lesions will be selected on the basis of (1) heat map color (probability of csPCa) and (2) lesion size.

When suspicious lesions are identified on both PCaVision and MRI, the TBx guided by each modality are performed in a single session. The biopsy order will be randomized using block randomization (with block sizes varying between six and ten) to prevent potential bias related to the order in which TBx procedures are performed.

If the same lesion is identified on both MRI and PCaVision, both an MRI TBx and a PCaVision TBx will be performed separately in the same session. It is up to the local physician whether routine SBx will be performed in addition to the study TBx.

### 3.1 Study procedures

#### 3.1.1 Prostate MRI

MRI will be performed with the patient in a supine position using a 3-T MRI scanner. Prebiopsy prostate MRI acquisition will be performed according to the most recent PI-RADS guidelines [22]. MRI sequences will include at least T1-weighted, T2-weighted, and diffusion-weighted imaging (DWI) and calculation of apparent diffusion coefficient (ADC) maps. Although recommended in PI-RADS v2.1, the use of dynamic contrast-enhanced (DCE) MRI is optional and is dependent on the local standard of care. This aligns with the EAU guidelines, which do not recommend multiparametric over biparametric MRI [6].

MRI scans will be evaluated blinded to the 3D mpUS results by specialized uroradiologists with prostate MRI experience and in accordance with the PI-RADS guidelines [22]. The quality of all MRI scans will be retrospectively evaluated by trained radiologists using PI-QUAL once the study is completed [23], [24], [25].

#### 3.1.2 3D mpUS with PCaVision

The investigational procedure consists of two components: (1) 3D mpUS imaging; and (2) PCaVision analysis.

For 3D mpUS imaging, a transrectal US probe is placed in the rectal cavity of the patient and secured to a probe fixture (Supplementary material). Patients will consecutively undergo 3D SWE, 3D B-mode, and 4D CEUS performed using two automated imaging scripts. For 4D CEUS, one 2.4-ml bolus of SonoVue (sulfur hexafluoride; Bracco Imaging S.p.A., Colleretto Graciosa, Italy) will be administered intravenously (off-label use). A 2-min recording in contrast-enhanced mode will be made following SonoVue administration. The entire 3D mpUS imaging acquisition process will take approximately 10 min. A clinically approved US system (LOGIQ E10; GE Healthcare, Milwaukee, WI, USA) will be used for the study.

The 3D mpUS images will be stored and automatically transferred to a computer immediately after acquisition for analysis using PCaVision software. Different analyses will be performed for each modality (B-mode, CEUS, and SWE), resulting in a 3D mpUS classifier parametric image heat map within 15 min.

Imaging for the first three to five patients for each investigator in each participating center will be performed under expert supervision to optimize adherence to the protocol. The results obtained for these patients will be incorporated in the final statistical analysis. During these supervised cases, the expert will be on-site to monitor study procedures, identify problems, and provide feedback on both 3D mpUS acquisition and interpretation of the PCaVision results. The expert will ensure that the results obtained are of sufficient quality and will certify the investigators. Training records will be maintained to document training of staff.

#### 3.1.3 Biopsy procedure and histological examination

A urologist will perform software-assisted fusion TBx or cognitive TBx, depending on the local practice in each participating center. MIM (MIM Software Inc., Cleveland, OH, USA) integrated elastic MRI/US fusion software is used for software-assisted fusion TBx. Each center is adequately trained on this system.

For each imaging modality (MRI and 3D mpUS), a maximum of two lesions will be targeted. Each lesion will be sampled using three biopsy cores. If both imaging techniques identify lesions to be biopsied in an individual patient, the TBx procedures are performed in a randomized order to prevent bias as already described. Every participating center will use a transperineal biopsy approach.

Individual TBx cores will be analyzed separately by a uropathologist for the presence of PCa (Gleason score), percentage tumor core involvement, and morphological patterns, including cribriform growth pattern and intraductal carcinoma, in accordance with the 2019 ISUP guidelines [26]. A work order instruction for the exact sample labeling of the biopsies and histopathological workup will be provided and agreed with each site. The pathologists interpreting TBx (and SBx if available) will be blinded to which imaging modality was used for the TBx.

### 3.2 Safety and risks

Adverse events are defined as any undesirable event experienced by a subject during the study, whether or not it is related to the (non-)investigational product or trial procedure. All adverse events reported by a participant or observed by the investigator or their staff will be recorded. All patients will receive the standard of care (MRI pathway), so the risk of diagnostic pathway compromise is negligible. The study has been approved by an accredited institutional review board (IRB) and is registered on ClinicalTrials.gov as NCT06281769.

#### 3.2.1 Premature termination of the study

MRI and TBx are already performed as part of routine clinical care. It is considered that the extra PCaVision TBx procedures carry minimal additional risk over routine biopsies. Therefore, the likelihood of premature termination of the study because of risk to participants is considered to be very low. A mandatory requirement is discussion of every serious adverse event with the accredited IRB. If severe adverse events (grade 3; Common Terminology Criteria for Adverse Events version 3.0) caused by 3D mpUS imaging of the prostate with contrast agent (SonoVue), the principal investigator can decide to terminate the study in consultation with the accredited IRB. After premature termination of the study, patients will still receive the standard clinical treatment.

### 3.3 Follow-up activities

The likelihood of adverse events associated with use of the contrast agent (SonoVue) is negligible. It is expected that any complications experienced by study participants will be adverse events related to the image acquisition or biopsy procedure. From clinical practice it is known that these events will manifest within 2 wk and will resolve within 6 wk. If there are no complications within 2 wk, the patient is considered to have completed the study. The study will be closed when all patients have completed the study, with a maximum follow-up of 6 wk if complications occur.

## 4 Statistical analysis

Analyses will compare histological findings from the two diagnostic pathways at a per-patient level, with differentiation between two data sets: (1) the total study population of all men included in the study to undergo PCaVision and MRI; and (2) a paired data set for patients with images of sufficient quality for both PCaVision and MRI. The second data set will be used in paired analyses comparing the diagnostic accuracy of the two imaging pathways.

### 4.1 Primary outcome analysis

The main outcome is comparison of the csPCa detection rate between the PCaVision and MRI pathways. We will calculate the difference in these detection rates and the corresponding 97.5% confidence interval. Because of the paired design of our study, we will use the approach of Liu et al [27], [28], [29], which accounts for correlation between the detection rates of the two pathways. A one-sided 2.5% equivalence test will be performed to formally evaluate whether the lower bound of the confidence interval will not exceed the noninferiority margin specified at an absolute difference in detection rate of 5%.

### 4.2 Secondary outcome analysis

As a secondary analysis, we will compare the proportion of men in whom TBx could have been safely omitted in the PCaVision pathway versus the MRI pathway. For the PCaVision pathway, this proportion is the number of men in whom there are no suspicious lesions to be biopsied according to the PCaVision results and for whom the MRI TBx and SBx (if performed) did not detect csPCa, divided by the total number of men in the study. Likewise, this proportion for the MRI pathway is the number of men in whom there are no suspicious lesions to be biopsied according to the MRI results and for whom the PCaVision TBx and SBx (if performed) did not detect csPCa, divided by the total number of men in the study. Given the fully paired design, the proportions will be tested for differences using the McNemar test (two-sided α of 5%). The approach of Liu et al [27], [28], [29], which accounts for the correlation, will be used to calculate a 95% confidence interval for the difference between these proportions.

We will also perform the primary analysis using different thresholds for csPCa. These analyses will use the same effect measures and statistical approach as in the previous analyses, and only the ISUP grade definition of PCa will change. The following alternative definitions (rather than GG ≥2) will be used: (1) GG ≥3; (2) GG ≥2 with cribriform growth and/or intraductal carcinoma; and (3) GG 1.

Both PCaVision and MRI can produce images of insufficient quality for diagnosis and TBx. Therefore, we will compare the proportion of men with images of insufficient quality between the PCaVision and MRI modalities. The same statistical approach for paired proportions will be used for this analysis [28].

PCaVision has inbuilt quality thresholds for CEUS signal input and motion. The software does not present a result if these thresholds are not met. The detection rate of csPCa using PCaVision will be evaluated by applying various increasing thresholds for image quality (Supplementary material). We will use the aforementioned statistical approach for paired proportions to compare detection rates.

### 4.3 Sample size calculation

The sample size calculation was based on demonstrating noninferiority of the detection rate for csPCa for the PCaVision pathway versus the MRI pathway using patients as the unit of analysis and data from the fully paired design. The csPCa detection rates according to MRI and PCaVision TBx are expected to be approximately 30%. These values are based on the detection rates for csPCa (GG ≥2) reported by Mannaerts et al [30] and Grey et al [31], which were 28% and 26% with 2D CUDI, and 29% and 30% with MRI, respectively (30, 31). The noninferiority margin is set to a 5% absolute difference in the detection rate. The expected proportion of discordant pairs is 10%. In the above-mentioned studies, the percentage discordant pairs was 10.1% [30] and 8.9% [31].

Our assumptions for calculation of the sample size were therefore the following: an expected detection rate of 30% for both imaging techniques, a noninferiority margin of 5%, percentage discordance of 10% between the imaging techniques, a one-sided type 1 error of 2.5% (as recommended by the European Medicines Agency), and power of 80%. This leads to a required total sample size of 350 patients who need to undergo both PCaVision and MRI with images of sufficient quality, followed by TBx if indicated. This sample size was calculated according to Liu et al [28] and Hintze [32].

For both imaging pathways, we expect that either PCaVision or/and MRI will yield image quality that is insufficient to perform TBx, or technical issues will arise during TBx for a proportion of men. The primary paired analyses will be use data for men for whom images of sufficient quality and TBx for both imaging techniques are available. The total loss of study patients because of insufficient image quality, technical issues, and trial logistics is expected to be up to 25% [33]. To compensate for this loss, extra patients will be included. The total sample size will therefore be 438 to obtain data for 350 evaluable patients for the paired analysis. As we would like to include patients from five clinical centers, the inclusion target per center is 80 patients (range 40–120 patients).

### 4.4 Interim analysis

An interim analysis for re-estimation of the sample size will be performed when the data for 50% of the planned total sample size are available for review. We will determine the csPCa prevalence in the cohort and recalculate the sample size while leaving all other parameters the same. We will also evaluate the number of images with insufficient quality. If more patients are needed according to one of these recalculations, the total sample size will be adjusted accordingly in consultation with the IRB. The total sample size will not be reduced as a result of any recalculation.

It is considered that 3D mpUS acquisition and potential additional biopsies according to PCaVision results will pose minimal risk to patients. Patients will receive the normal standard of care from their own treating physician, so no interim analysis of safety will be performed.

## 5 Summary

This prospective multicenter trial will compare the diagnostic accuracy of a novel AI-based imaging pathway to a reference standard (MRI) for PCa detection. The current standard faces challenges related to the limited availability of MRI and the need for skilled radiologists. 3D mpUS with CAD has potential to mitigate these issues given its straightforward user interface and minimal training requirements for interpretation of results. However, it is important to acknowledge several limitations of our study design.

The primary limitation of our study is the choice of MRI as the reference standard. Owing to significant variability in MRI sensitivity across centers, the detection rates identified in our study may not accurately represent the true sensitivity for csPCa detection.

While inclusion of mandatory SBx in our study protocol could provide a more comprehensive assessment of the sensitivity of the two imaging modalities, it is crucial to acknowledge that SBx has limitations. SBx not only offers an imperfect representation of the actual presence of PCa but is also increasingly less preferred because of a higher risk of overdiagnosis and greater discomfort for patients [7], [8]. In an ideal scenario, template biopsy would serve as the gold standard for comparison. However, this approach presents logistic challenges and is considered less patient-friendly.

Despite these obstacles, we argue that use of MRI as the reference standard in our study offers the most accurate representation of both current and future clinical practice. This confidence is further reinforced by the fact that all participating centers in our study use high-quality MRI equipment and manage substantial patient volumes.

The potential influence of biopsy target inaccuracies on the study outcome must be acknowledged. Even though three TBx cores are taken per lesion, suspicious lesions based on one of the modalities can be missed. However, these target inaccuracies exist for both modalities and are a reflection of daily practice. In all centers, the biopsies will be conducted by dedicated urologists with experience in performing transperineal TBx.

The fully paired design is a valid and efficient approach, but it carries an inherent risk of the two diagnostic procedures influencing each other as some inadvertent interference may occur [34]. To address this risk, lesion identification will be performed by separate, blinded investigators and we will randomize the TBx order if both PCaVision and MRI identify suspicious lesions in an individual patient.

Furthermore, the study allows participating centers to maintain their MRI protocol (multiparametric or biparametric MRI) and reflects MRI practices in high-volume centers in The Netherlands. While this might be perceived as introducing variability, the literature shows that there is no evidence yet that multiparametric MRI has a better PCa detection rate over biparametric MRI [35].

Similarly, the decision to grant participating centers the autonomy to opt for either software fusion or a cognitive approach for TBx may be considered a potential source of bias. However, it is crucial to emphasize that for each patient, the same targeting strategy will be used for both imaging modalities. In addition, existing evidence does not show superiority of software fusion over cognitive methods in targeting PCa [36].

In conclusion, these methodological decisions underscore our commitment to capturing the diversity of clinical practices. These intentional variations, while introducing certain limitations, enhance the relevance of the study to real-world clinical practice and will contribute to the ongoing debate surrounding PCa diagnostic pathways.

This clinical validation trial will provide evidence on the clinical performance of a CAD pathway for PCa using PCaVision in comparison to the standard MRI pathway. If this CAD pathway proves to be sufficiently accurate in identifying csPCa, it holds great potential as an efficient and accessible alternative to the established MRI pathway.

  ***Author contributions:*** Daniel L. van den Kroonenberg had full access to all the data in the study and takes responsibility for the integrity of the data and the accuracy of the data analysis.

  *Study concept and design*: van den Kroonenberg, Jager, Garrido-Utrilla, Reitsma, Postema, Beerlage, Oddens.

*Acquisition of data*: van den Kroonenberg, Jager.

*Analysis and interpretation of data*: van den Kroonenberg.

*Drafting of the manuscript*: van den Kroonenberg.

*Critical revision of the manuscript for important intellectual content*: van den Kroonenberg, Jager, Garrido-Utrilla, Reitsma, Postema, Beerlage, Oddens.

*Statistical analysis*: van den Kroonenberg, Reitsma.

*Obtaining funding*: Beerlage, Oddens.

*Administrative, technical, or material support*: Garrido-Utrilla, Reitsma.

*Supervision*: Beerlage, Oddens.

*Other*: None.

  ***Financial disclosures:*** Daniel L. van den Kroonenberg certifies that all conflicts of interest, including specific financial interests and relationships and affiliations relevant to the subject matter or materials discussed in the manuscript (eg, employment/affiliation, grants or funding, consultancies, honoraria, stock ownership or options, expert testimony, royalties, or patents filed, received, or pending), are the following: Arnoud W. Postema, Auke Jager, and Johannes B. Reitsma are scientific advisors for Angiogenesis Analytics, for which they receive compensation. Anna Garrido-Utrilla is a full-time employee of Angiogenesis Analytics. Harrie P. Beerlage is chief of the clinical board for Angiogenesis Analytics. Daniel L. van den Kroonenberg and Jorg. R. Oddens have nothing to disclose.

  ***Funding/Support and role of the sponsor:*** This work was supported by Angiogenesis Analytics and the Transition program of the European Innovation Council (#101057919 PCaVision). The sponsors had a role in the design and conduct of the study and preparation of the manuscript.
