# Supplementary material for: Clinical Validation of Multiparametric Ultrasound for Detecting Clinically Significant Prostate Cancer Using Computer-Aided Diagnosis: A Direct Comparison with the Magnetic Resonance Imaging Pathway
Source: Eur Urol Open Sci. 2024 Jul 1;66:60–6. doi: 10.1016/j.euros.2024.06.012 (PMC11267110; doi:10.1016/j.euros.2024.06.012)
Supplement: Supplementary Data 1 [file mmc1.docx]

## Supplementary Material

# INVESTIGATIONAL PRODUCT

Below is a summary of the information on the product that analyses the 3D mpUS images which consists of a computer module with PCaVision™ that will be used in this study.

- The computing module is an off-the-shelf embedded computer which is enclosed in a medical-certified housing and is attached to the ultrasound machine. A wired network cable connects the computer module with the ultrasound machine enabling PCaVision™ to communicate and acquire data.
- PCaVision™ is the Image Analysis and Viewing Software for analysing 3D contrast enhanced and other modality ultrasound data of the prostate.

# NON-INVESTIGATIONAL PRODUCT

In the study, the following non-investigational products will be used:

- Probe fixture
- Ultrasound contrast agent Sonovue® (sulphur hexafluoride, Bracco Imaging S.p.A., Colleretto Giacosa, Italy)
- 3D ultrasound machine LOGIQTM E10 with RIC 5-9D 3D transrectal probe (GE Heathcare, Milwaukee, WI, USA)

### Probe fixture

The probe fixture is a CE-marked class I medical device. An extensive risk assessment was performed, and all potential risks were deemed acceptable. Ex vivo testing and analysis concluded that the probe fixture provides all desired functionalities.

The probe fixture is a device that is intended to provide fixation of a transrectal ultrasound imaging probe during prostate diagnostic imaging. The device is not intended to be used other than for ultrasound imaging, such as minimally invasive puncture procedures. The probe fixture will assist the ultrasound operator during image acquisition during this trial. The fixture can be attached to a DIN rail on the operating table. The ultrasound probe can be placed in the fixture and inserted. Once the probe is in the desired position the fixture can be fixed in place. Fixing the probe releases the operator from holding the probe during the scanning procedure, while providing stable image acquisition.

The probe fixture should be used by trained medical personnel only. The probe fixture features a central single-point locking mechanism to rapidly lock the fixture position without transducer migration (Supplementary Figure 1).

### Ultrasound contrast agent

Sonovue® (sulphur hexafluoride, Bracco Imaging S.p.A., Colleretto Giacosa, Italy)

Information extracted from the European public assessment report (EPAR) for SonoVue (available from http://www.ema.europa.eu/ema/). The EPAR was last updated on 14/09/2021.

SonoVue is a medicine that contains the active substance sulphur hexafluoride (a gas). It is available as a kit including one vial of gas and powder and one prefilled syringe containing 5 ml of solvent. When made up into a solution, SonoVue contains sulphur hexafluoride gas as ‘microbubbles’ in suspension in a liquid. SonoVue is for diagnostic use only. It is a contrast agent (it helps make internal body structures visible during imaging tests). SonoVue is used in tests that measure how ultrasound travels within the body because it improves the ability of the blood to create an echo. The medicine can only be obtained with a prescription. SonoVue should only be used by doctors who have experience in diagnostic ultrasound imaging. It is injected intravenously (into a vein) before the test is carried out, as a 2- or 2.4-ml dose depending on which test is being carried out. The dose can be repeated. The active substance in SonoVue, sulphur hexafluoride, is a gas that is not soluble in the blood. When SonoVue is made up into a suspension, the gas is trapped in tiny bubbles called microbubbles. After injection, the microbubbles travel in the blood, where they reflect ultrasound waves more than the surrounding tissues. This helps to enhance the results of tests that rely on measuring ultrasound, such as echocardiography and Doppler tests. The gas is removed naturally from the body through the lungs.

The most common side effects with SonoVue (seen in between 1 in 100 and 1 in 1,000 patients) are headache, paraesthesia (unusual sensations like pins and needles), dizziness, dysgeusia (taste disturbances), flushing (reddening of the skin), pharyngitis (sore throat), nausea (feeling sick), abdominal pain, pruritus (itching), rash, back pain, chest discomfort, reactions at the injection site, feeling hot and raised blood sugar levels. For the full list of all side effects reported with SonoVue, see the package leaflet. SonoVue must not be used in patients known to have right-to-left shunts (abnormal movement of blood within the heart), severe pulmonary hypertension (high blood pressure in the pulmonary artery, the blood vessel that leads from the heart to the lungs), uncontrolled hypertension (high blood pressure) or adult respiratory distress syndrome (severe fluid build-up in both lungs). SonoVue must also not be used together with the medicine dobutamine (used for heart failure) in patients for whom dobutamine is not suitable. For the full list of restrictions, see the package leaflet. The CHMP decided that SonoVue’s benefits are greater than its risks and recommended that it be given marketing authorisation. The European Commission granted a marketing authorisation valid throughout the European Union for SonoVue on 26 March 2001.

Ultrasound contrast agent Sonovue® (sulphur hexafluoride, Bracco Imaging S.p.A., Colleretto Giacosa, Italy) is registered for use in:

- Echocardiography
- Doppler of macrovasculature
- Doppler of microvasculature
- Ultrasonography of excretory urinary tract

### 3D ultrasound machine

LOGIQ^TM^ E10 (GE Heathcare, Milwaukee, WI, USA) with RIC 5-9D 3D transrectal probe (GE Healtcare, Chicago, USA)

Information extracted from the Technical Publications

Direction 5750001-1EN Rev. 3.

(available from https://customer-doc.cloud.gehealthcare.com/#/cdp/dashboard)

The document was last updated on 15/06/2019.

Medical ultrasound images are created by computer and digital memory from the transmission and reception of mechanical high-frequency waves applied through a transducer. The mechanical ultrasound waves spread through the body, producing an echo where density changes occur. For example, in the case of human tissue, an echo is created where a signal passes from an adipose tissue (fat) region to a muscular tissue region. The echoes return to the transducer where they are converted back into electrical signals. These echo signals are highly amplified and processed by several analog and digital circuits having filters with many frequency and time response options, transforming the high-frequency electrical signals into a series of digital image signals which are stored in memory. Once in memory, the image can be displayed in real-time on the image monitor. All signal transmission, reception and processing characteristics are controlled by the main computer. By selection from the system control panel, the user can alter the characteristics and features of the system, allowing a wide range of uses, from obstetrics to peripheral vascular examinations. Transducers are accurate, solid-state devices, providing multiple image formats. The digital design and use of solid-state components provides highly stable and consistent imaging performance with minimal required maintenance. Sophisticated design with computer control offers a system with extensive features and functions which is user-friendly and easy to use.

LOGIQ^TM^ E10 (GE Heathcare, Milwaukee, WI, USA) complies with regulatory requirements of the following European Directive 93/42/EEC concerning medical devices. First CE Marked in 2018. The LOGIQ E10 is intended for use by a qualified physician for ultrasound evaluation.

Specific clinical applications and exam types include:

- Fetal/Obstetrics
- Abdominal (includes Renal, Gynecology/Pelvic)
- Pediatric
- Small Organ (Breast, Testes, Thyroid)
- Neonatal Cephalic
- Adult Cephalic
- Cardiac (Adult and Pediatric)
- Peripheral Vascular
- Musculo-skeletal Conventional and Superficial
- Urology (including Prostate)
- Transrectal
- Transvaginal
- Transesophageal

Intraoperative (Vascular) Image Acquisition is for diagnostic purposes, including measurements on acquired images.

**Supplementary figures**

Supplementary figure 1: Probe fixture

**Supplementary Table**Supplementary table 1: PCaVision™ image quality threshold

| **Quality parameter** | **Description** | **Loose*** | **Strict*** |
| --- | --- | --- | --- |
| CEUS signal | Minimum peak intensity value (dB) | 43 | 50 |
| Patient motion | Minimum IoU (intersection over union) of B-mode prostate contours | 0.60 | 0.70 |
|  | Maximum root mean square magnitude of detected motion vectors (mm) | 8.1 | 3.0 |

*Loose criteria are default and used for primary outcome analysis. A combination of stricter criteria is used for secondary outcome analysis.

CEUS: contrast-enhanced ultrasound; dB: decibel; mm: millimetre.
